# Cancer patients’ willingness to answer survey questions about life expectancy

**DOI:** 10.1007/s00520-012-1477-9

**Published:** 2012-05-11

**Authors:** L. J. Mackenzie, M. L. Carey, R. W. Sanson-Fisher, C. A. D’Este, A. E. Hall

**Affiliations:** 1 Priority Research Centre for Health Behaviour, School of Medicine and Public Health, Faculty of Health, University of Newcastle, Callaghan, New South Wales 2308 Australia; 2 Priority Research Centre for Gender, Health & Ageing; Priority Research Centre for Health Behaviour; Centre for Clinical Epidemiology and Biostatistics, University of Newcastle, Callaghan, New South Wales 2308 Australia; 3 Graduate School of Medicine, Kyoto University, Breast Surgery, Kyoto University Hospital, 54 Kawahara-cho Shogoin, Sakyo-ku, Kyoto, Japan

**Keywords:** Cancer, Patient-centered care, Patient preference, Prognosis

## Abstract

**Purpose:**

This study aimed to determine the proportion and characteristics of radiation oncology outpatients who were willing to answer questions about their life expectancy.

**Methods:**

A cross-sectional patient self-report survey was conducted using touch screen computers in Australian radiation oncology treatment centers. The primary outcome was the respondent’s willingness to complete a survey subsection about life expectancy. Demographic and disease characteristics were also collected, and level of anxiety and depression was assessed using the Hospital Anxiety and Depression Scale.

**Results:**

Of the 469 oncology outpatients who completed the survey, 327 (70 %; 95 % CI, 65 %, 74 %) indicated that they were willing to answer questions about life expectancy. Being female (*p* < 0.001), older (*p* < 0.05), born in Asia (*p* < 0.05), and being diagnosed with cancer types other than breast and prostate cancer (*p* < 0.01) were associated with lower odds of answering life expectancy questions.

**Conclusions:**

The opportunity to opt-out of survey questions about sensitive issues such as life expectancy is a feasible method for accessing important information about patient preferences while minimizing burden. Further research may be needed to improve acceptability of life expectancy research to some patient groups.

## Introduction

A cancer diagnosis is often associated with a reduced life expectancy [1]. Therefore oncologists are often faced with the intimidating task of communicating this bad news to patients and their families [2]. Although communication training is provided in medical schools, clinicians have reported feeling inadequately trained in breaking bad news [3]. Variation in individual patient preferences for life expectancy disclosure compounds the complexity of this task for clinicians [3, 4]. Additionally, there is limited evidence about patient disease and demographic characteristics associated with different preferences [5]. Although guidelines based on consensus views have been developed to assist clinicians with the task of life expectancy disclosure [6, 7], it remains critical to build empirical evidence about how different methods of disclosure impact on patient psychosocial outcomes [2].

Although there is a need to build the evidence base regarding prognostic communication [5], ethical concerns about the potential burden of this research have been raised [8]. The development of a patient-centered methodological approach to assessing patients’ preferences for life expectancy disclosure may prove successful for increasing representative research output, while also minimizing the burden of research to patients in accordance with their preferences for involvement. This approach involves embedding a set of optional life expectancy questions within a larger patient experience survey and giving patients the chance to “opt-out” of these questions. It was thought that this may help to minimize patient burden, while also maximizing overall consent rates and representativeness [9]. Importantly, this approach would also allow identification of patient characteristics relating to a willingness to answer these questions, helping to improve our understanding of acceptability of the topic of life expectancy to different cancer patient groups [5].

## Aims and hypotheses

This research aimed to determine (1) the proportion of cancer patients willing to answer survey questions about their life expectancy, and (2) examine disease and demographic factors associated with a willingness to answer these questions. We expected that females would be more likely than males to answer life expectancy questions, based on past findings suggesting females want more detailed information about cancer [10]. Based on previous research into cancer patients’ preferences for prognosis communication, it was expected that patients perceiving a shorter survival time would be less likely to complete life expectancy questions than those perceiving a longer survival time [11]. Similarly, we expected that patients reporting that their treatment had curative intent would be more willing to complete the life expectancy questions than those perceiving that their treatment was with palliative intent. It has also been suggested that willingness to participate in research trials may be linked to psychological distress; however, findings are mixed [2, 5, 12]. We expected that respondents born in some Asian and European regions may be less likely to complete life expectancy questions [4, 5], and that younger respondents would be more willing to answer the life expectancy questions [5, 11].

## Patients and methods

### Ethical standards

All human research was approved by the University of Newcastle HREC and the New South Wales Population and Health Services Research Ethics Committee. Research governance authorization was also sought and obtained from the participating hospitals.

### Patients

Cancer outpatients were recruited from four metropolitan radiation therapy treatment centers between February 2010 and December 2010. The four treatment centers were attached to large public teaching hospitals located in high socio-economic status areas of Sydney, Australia. Patients were eligible for inclusion if they were aged 18 years or older; had a cancer diagnosis; were attending radiation therapy treatment as an outpatient, and understood sufficient English to complete the survey. Patients who were attending the clinic for the first time were excluded.

### Procedure

A research assistant (RA) approached patients waiting for their radiation therapy treatment appointment. Participants were provided with a written information statement about the study. The RA explained that the survey contained questions about quality of care, coping with cancer, and an optional section about life expectancy. The RA then sought informed consent from eligible patients after indicating that the patient could choose not to complete the section on life expectancy. Once informed consent was obtained, patients completed the survey in the waiting room using a portable touch screen computer. Digivey software (CREOSO Corporation, Phoenix, Arizona) was used to program the survey, which was administered using a Dell Latitude XT2 touch screen computer. The use of touch screen computer surveys in oncology settings has been previously found to be acceptable to cancer patients [13].

### Measures

The following modules were embedded within a larger 10–15-min survey:

#### Outcome measure

The primary outcome was patients’ willingness to complete life expectancy questions. The introduction to this section of the survey read “The following questions ask for your views about your life expectancy. This will provide information that may help to improve services for cancer patients.” Participants were asked to indicate whether or not they were willing to complete questions about their life expectancy by either selecting “I am willing to complete this section of the survey” or “Please skip to the next section of the survey”. If participants initially chose to complete the life expectancy section, but then changed their mind, they could use the “BACK” navigation button on the survey screen to return to the introductory screen for the life expectancy section. This then allowed participants to skip the life expectancy section and any previous responses were deleted.

#### Explanatory measures

Demographic and disease data on age, gender, diagnosis, country of birth, time since diagnosis, number of outpatient appointments, number of oncology appointments, and treatment aim were collected via the self-report survey. The Hospital Anxiety and Depression Scale (HADS) was used to assess anxiety and depression. The HADS contains two seven-item subscales each producing a score between 0 and 21 indicating normal (0–7), mild (8–10), moderate (11–14), or severe (15–21) levels of anxiety, and/or depression [14]. There is evidence that this measure is both reliable and valid in a cancer patient population [15], and produces comparable results when it is administered via paper and pencil or via touch screen computers [16]. Although various thresholds have been used in the literature to identify caseness [17], a subscale score of 11 or more can be indicative of clinically significant levels of anxiety and/or depression [14]. This threshold was used to classify respondents having clinically significant depression or anxiety.

### Statistical analysis

The proportion of participants willing to answer survey questions about their life expectancy was estimated with a 95 % confidence interval. Disease and demographic factors hypothesized as being associated with a willingness to answer these questions were examined using univariate and multiple logistic regression analyses. Because of small numbers of rarer cancer types, the cancer type variable was collapsed across low incidence categories to give the following “breast,” “prostate”, and “other” for univariate analysis. Variables of interest included: age category, sex, region of birth, clinically significant anxiety, clinically significant depression, cancer type, and perceived palliative treatment aim. Variables with a *p* value of 0.2 or less for univariate analysis were included in a multiple logistic regression model, and the backward stepwise method was used to remove variables with a *p* value of 0.1 or greater on the likelihood ratio test. Odds ratios with 95 % confidence intervals were calculated for univariate and multiple regression models and a significance level of 5 % was used. Recruitment site (hospital) was included in the multiple regression analysis to control for between site differences in patient characteristics. Analyses were undertaken using STATA version 11.2.

### Sample size

This study aimed to recruit a total of 450 patients from four hospital sites, which would allow us to obtain prevalence estimates with 95 % CI’s with ±5 % of the point estimate. This sample size would also allow us to detect differences of 15 % in characteristics between the groups who opt-in and who opt-out of the life expectancy section with a 5 % significance level and 80 % power.

## Results

Of the 785 patients screened for eligibility, 126 did not meet the eligibility criteria. Of the 659 patients who were invited to join the study, 570 (86 %) consented to participate in the survey, of whom 82 % (n=469; 71 % of all eligible participants) completed the survey in its entirety. Data was not available for participants who consented but were unable to complete the survey due to time limitations. Participants were 242 males and 227 females, with a mean age of 61.5 years (SD = 13.2, median = 62.9; Q1, Q3, 52.4, 70.2*).* At the time of recruitment, respondents were a mean of 85.8 weeks since diagnosis (SD = 169.1, median = 28.7; Q1, Q3, 16.2, 57.2), and had attended a mean of 11.6 outpatient radiation therapy appointments (SD = 10.2, median = 9; Q1, Q3, 4, 18). Respondents reported that their most recent primary cancer diagnosis was breast (28 %), prostate (22 %), head and neck (9.6 %), colorectal (bowel; 5.3 %), brain (4.3 %), lung (4.0 %), melanoma (3.4 %), non-Hodgkin’s lymphoma (3.2 %), other cancers (17 %), and 2.1 % did not know. Sixty-nine percent of respondents were Australian born.

Three hundred and twenty-seven (70 %, 95 % CI, 65 %, 74 %) of the 469 participants who completed the survey indicated that they were willing to answer questions about life expectancy. Overall, this meant that 50 % of all eligible participants completed the survey questions about life expectancy. Table 1 outlines the results of the univariate and multiple logistic regression analysis assessing characteristics associated with willingness to complete the life expectancy questions. Following univariate analysis, clinically significant anxiety (*p* = 0.3) and perceived palliative treatment aim (*p* = 0.5) were excluded from the model (see Table 1). Remaining variables entered into the multiple logistic regression analysis included age group, gender, cancer type, region of birth, and depression. Compared to the youngest age group (18–49 years) those aged 60–69 years and 70 years or more had significantly lower odds of answering the life expectancy questions (see Table 1). Females had significantly lower odds of answering the life expectancy questions than males. Participants born in Asia had lower odds of answering the life expectancy questions than Australian-born participants, and a similar trend was seen for European-born participants (although marginally non-significant). Compared to participants with breast cancer, participants with “other cancers” (i.e., not breast or prostate cancer) had significantly lower odds of completing the life expectancy questions. Having clinically significant depression according to the HADS was not found to be significantly associated with the outcome of interest in the multiple regression model.

**Table 1:** Univariate and multiple logistic regression of characteristics of 469 participants completing the patient views survey

	Willing to complete life expectancy questions *n* (row %)	Unadjusted OR (95 % CI)	Adjusted OR (95 % CI)	Likelihood ratio *χ* ^2^(df), *p*
Hospital				4.0 (3), *p* = 0.2663
Site 1	121 (70 %)	1	1	
Site 2	90 (65 %)	0.8 (0.4–1.2)	0.8 (0.5–1.3)	
Site 3	51 (68 %)	0.9 (0.5–1.6)	1.0 (0.5–1.9)	
Site 4	65 (77 %)	1.4 (0.8–2.6)	1.5 (0.8–2.8)	
Age group				10.7 (3), *p* = 0.0137
18–49	72 (77 %)	1	1	
50–59	73 (72 %)	0.7 (0.4–1.4)	0.6 (0.3–1.3)	
60–69	104 (68 %)	0.6 (0.3–1.1)	0.4 (0.2–0.8)	
70+	78 (64 %)	0.5 (0.3–1.0)	0.4 (0.2–0.7)	
Sex				13.9 (1), *p* < 0.0002
Male	179 (74 %)	1	1	
Female	148 (65 %)	0.7 (0.4–1.0)	0.3 (0.2–0.6)	
Region of birth				10.8 (4), *p* = 0.0291
Australia	237 (73 %)	1	1	
UK and Ireland	26 (67 %)	0.7 (0.4–1.5)	0.7 (0.3–1.4)	
Asia	19 (58 %)	0.5 (0.2–1.0)	0.4 (0.2–0.8)	
Europe	20 (57 %)	0.5 (0.2–1.0)	0.5 (0.2–1.0)	
Other	25 (66 %)	0.7 (0.3–1.4)	0.5 (0.2–1.1)	
Palliative treatment aim				
Yes	43 (73 %)	1		
No	263 (69 %)	1.2 (0.7–2.2)		
Cancer type				13.4 (2), *p* = 0.0012
Breast	99 (74 %)	1	1	
Prostate	79 (75 %)	1 (0.6–1.9)	0.5 (0.2–1.1)	
Other^a^	149 (65 %)	0.6 (0.4–1.0)	0.3 (0.2–0.6)	
Clinically significant anxiety^b^				
Yes	44 (58 %)	0.8 (0.4–1.3)		
No	281 (70 %)	1		
Clinically significant depression^b^				1.0 (1), *p* = 0.3151^c^
Yes	15 (65 %)	0.6 (0.3–1.3)		
No	310 (71 %)	1		

Observations within each variable may not add to the total due to missing values

^a^Including brain, colorectal, head and neck, lung, non-Hodgkin’s lymphoma, and other cancer types

^b^Assessed using the Hospital Anxiety and Depression Scale

^c^Eliminated during backwards stepwise multiple logistic regression analysis

## Discussion

In the current study, 70 % of participants who completed the survey (and 50 % of all eligible patients) were willing to answer a subset of questions about their life expectancy. Previous research into patients’ preferences for life expectancy information has yielded similar consent rates. A large USA-based interview study achieved a consent rate of approximately 70 % when assessing the views of 638 advanced cancer patients (recruited from outpatient clinics at seven hospital sites) about whether end of life care discussions had occurred [18]. Australian survey research into cancer patients’ prognostic communication preferences have obtained consent rates of 61 % [19]; while interview/focus group studies with palliative care patients have obtained consent rates of up to 83 % [20]. However, recruitment for these studies was conducted through oncologists or palliative care services, and poor consent rates among oncologists or community nurses may have introduced response bias [19].

Our findings indicating that older participants had lower odds than younger participants of answering questions about life expectancy appear to be consistent with past research. Kaplowitz and colleagues [11] found that older people were significantly less likely to request and to be given prognosis information. Similarly, a review by Fujimori and colleagues reported that younger patients were more likely to express a desire for more prognosis information than older patients [5]. Younger patients may be more likely to have dependent children and be willing to discuss life expectancy information for planning purposes [21]. This may also reflect changes in patient attitudes, preferences, and expectations over time towards increased involvement in cancer treatment decision making [22].

The increased willingness to answer questions on life expectancy among males was contrary to our expectations. It has been reported that women diagnosed with cancer are more likely to want more detailed general information about cancer than men [10]. A study of response rates to an epidemiological survey involving 25,000 participants found that consent to review medical records higher in males older than 50 than females over 50 [23]. Other research has found that men were significantly more likely to be given a quantitative life expectancy estimate than women [11]. Taken together, these findings may suggest that males are more likely to be willing to be involved in research about personal or potentially sensitive issues than females.

Our findings showed that Australian-born participants had higher odds of completing life expectancy questions than respondents born in the Asian region, and marginally non-significantly higher odds than those born in Europe. Previous reports have indicated that culture may influence patients’ preferences for information about life expectancy prognosis discussions [5]. A recent review of patients’ preferences for life expectancy communication reported that studies conducted in Asian countries have reported that fewer than 30 % of patients wish to know about life expectancy, while approximately 60 % of Westerners do [4, 5]. Regional variations in physician views and practices surrounding disclosure of palliative illness status in Europe, Latin America, and Canada have also been reported [24]. These differences may be related to beliefs about the potential impact that awareness of a poor life expectancy estimate may have on patient hope, and consequentially on outcomes. Therefore, the present findings may be reflective of cultural attitudes towards discussion of life expectancy. However, a recent qualitative study looking at communication preferences in migrants to Australia with Greek-, Arabic-, and Chinese-speaking backgrounds, suggests that these migrant groups are possibly more likely than Anglo Australian patients to prefer to have access to prognostic information [25]. It may be that a willingness to answer questions about life expectancy is not comparable to a patient preference to have access to prognostic information. It is also possible that responses regarding willingness to answer life expectancy questions in the current study may have been influenced by family members who may have been accompanying them in the waiting room during survey completion. Further exploration of discordance between patient and family preferences may be warranted in Australian and international settings. However, it does seems likely that some level of cultural variation in patient preferences for answering life expectancy questions exists and further exploration of how this relates to patients preferences for life expectancy disclosure may be warranted. This is particularly pertinent given that individuals without adequate English language to complete the survey were excluded from this study. Future studies should extend these findings to culturally and linguistically diverse communities [25].

Prior research has indicated that patients who identified themselves as having a shorter survival time may be less likely to want, request, and receive life expectancy information compared to those perceiving longer survival time [11]. It has also been suggested that an increased physical burden of cancer may be associated with a preference to have less involvement in cancer care decision making [10]. The current study found no association between patients’ perceived treatment aim and willingness to answer the life expectancy questions. However, individuals in the present study diagnosed with cancers with high 5-year survival rates (i.e., breast or prostate) [1] were more willing than those with other cancers to complete life expectancy questions. It is possible that the greater willingness among breast and prostate cancer patients was linked to perceived length of life. This would appear consistent with the findings of Kaplowitz and colleagues [11]. For instance, increased rates of distress in some poorer prognosis cancer types such as of the lung and brain have been suggested to be associated with feelings of “doom” [26]. This finding warrants further exploration, as the preferences of patients with less common cancers tend to be under reported in current consensus guidelines.

### Limitations

This research compared the characteristics of survey participants who were and were not willing to answer a subset of questions about their life expectancy. However, as part of the consent process potential participants were made aware of the optional section about life expectancy, and potential respondents (for whom demographic information is not available) may have opted out at this point. Additionally, although the current study achieved high consent rates to the initial survey (87 %), only 70 % of all eligible patients completed the entire survey in the time available. Once again, demographic information is not available for participants with incomplete surveys, meaning comparisons between survey completers and non-completers are not possible. Given that 70 % of eligible participants completed the survey in its entirety and 70 % of these respondents were willing to answer questions about their life expectancy, overall, 50 % of all eligible participants completed the life expectancy questions. Although this overall consent rate is comparable to other research [27], it may limit the external validity of the study results.

All patient demographic and disease data was collected via patient self-report, meaning accuracy for some items may be questioned [28]. Accuracy of self-reported cancer history validated against medical records and cancer registry data has been found to be high [29], with high sensitivity in cancer outpatient samples [30].

### Implications

There remains a need for high-quality research in the area of life expectancy communication. However, this research needs to minimize the risk of psychological distress to patients while also maximizing the representativeness of samples consulted. This approach to empowering patients to decide whether or not to answer research questions of this nature, rather than having access to patients restricted by clinical gatekeepers, requires a balancing of the ethical principles of beneficence and autonomy [31]. The current study found high consent rates to both the initial survey and also to the life expectancy questions embedded within the main survey, which resulted, in an overall acceptable consent rate. This suggests that this patient-centered approach to researching this sensitive topic was both feasible and acceptable, but the degree of acceptability varied across different subgroups. Further research may be needed to identify how to improve acceptability of this research to subgroups including those who are female, aged 60 years or over, diagnosed with less common cancer types, and Australian migrants from Asian regions [5, 22].

## Conclusions

Giving cancer patients the opportunity to opt out of questions about a sensitive issue is a feasible and acceptable option for accessing important information about patient preferences. This method also promotes greater autonomy than clinician-determined methods of access to patients for these types of surveys. Further research may be needed to identify approaches to improve acceptability of research on life expectancy discussions with cancer patients who are older, female, diagnosed with less common cancer types, and who are born in Asia.
